# Engineered limestone: closing the carbon loop through geo- and bio-inspired mineralization

**DOI:** 10.1093/nsr/nwag485

**Published:** 2026-08-18

**Authors:** Zhichao Liu, Meicheng Zhao, Shuguang Hu, Xiaoyu Yang, Fazhou Wang

**Affiliations:** State Key Laboratory of Silicate Materials for Architectures, Wuhan University of Technology, China; School of Materials Science and Engineering, Wuhan University of Technology, China; State Key Laboratory of Silicate Materials for Architectures, Wuhan University of Technology, China; School of Materials Science and Engineering, Wuhan University of Technology, China; State Key Laboratory of Silicate Materials for Architectures, Wuhan University of Technology, China; State Key Laboratory of Silicate Materials for Architectures, Wuhan University of Technology, China; State Key Laboratory of Silicate Materials for Architectures, Wuhan University of Technology, China

## Abstract

This Perspective introduces engineered limestone, a geo- and bio-inspired strategy that uses industrial carbon dioxide to harden reactive minerals and wastes into durable construction products, helping close the carbon loop.

In nature, limestone is generated by the calcium-silicate cycle. The process is initiated by the dissolution of CO_2_ in water, which then weathers calcium-bearing silicate rocks to form aqueous calcium and dissolved inorganic carbon species. Following transportation of this solution through the aqueous environment, ultimate precipitation of stable carbonate minerals occurs in the form of limestone (Fig. 1a) [1]. While this geological pathway has led to the storage of huge amounts of carbon for millions of years, it takes place too slowly to be effectively employed to meet the limestone demands of modern society or as a solution to the problem created by excess CO_2_ in the atmosphere. The cement industry has engineered the calcium-silicate cycle to perform the reverse process. In this industry, natural limestone is calcined with siliceous and aluminous raw materials at high temperatures to form calcium silicate clinkers, such as C_3_S and β-C_2_S, along with large quantities of CO_2_ (Fig. 1b) [2]. Therefore, the engineered carbonate–silicate transformation employed for producing cement creates an open carbon loop in which CO_2_ captured naturally over a long period is released into the environment in a comparatively short time. The phrase ‘closing this loop’, as discussed here, does not imply full carbon neutrality by itself; rather, it means reincorporating industrial CO_2_ emissions into stable mineral phases and useful construction products.

**Figure 1.**
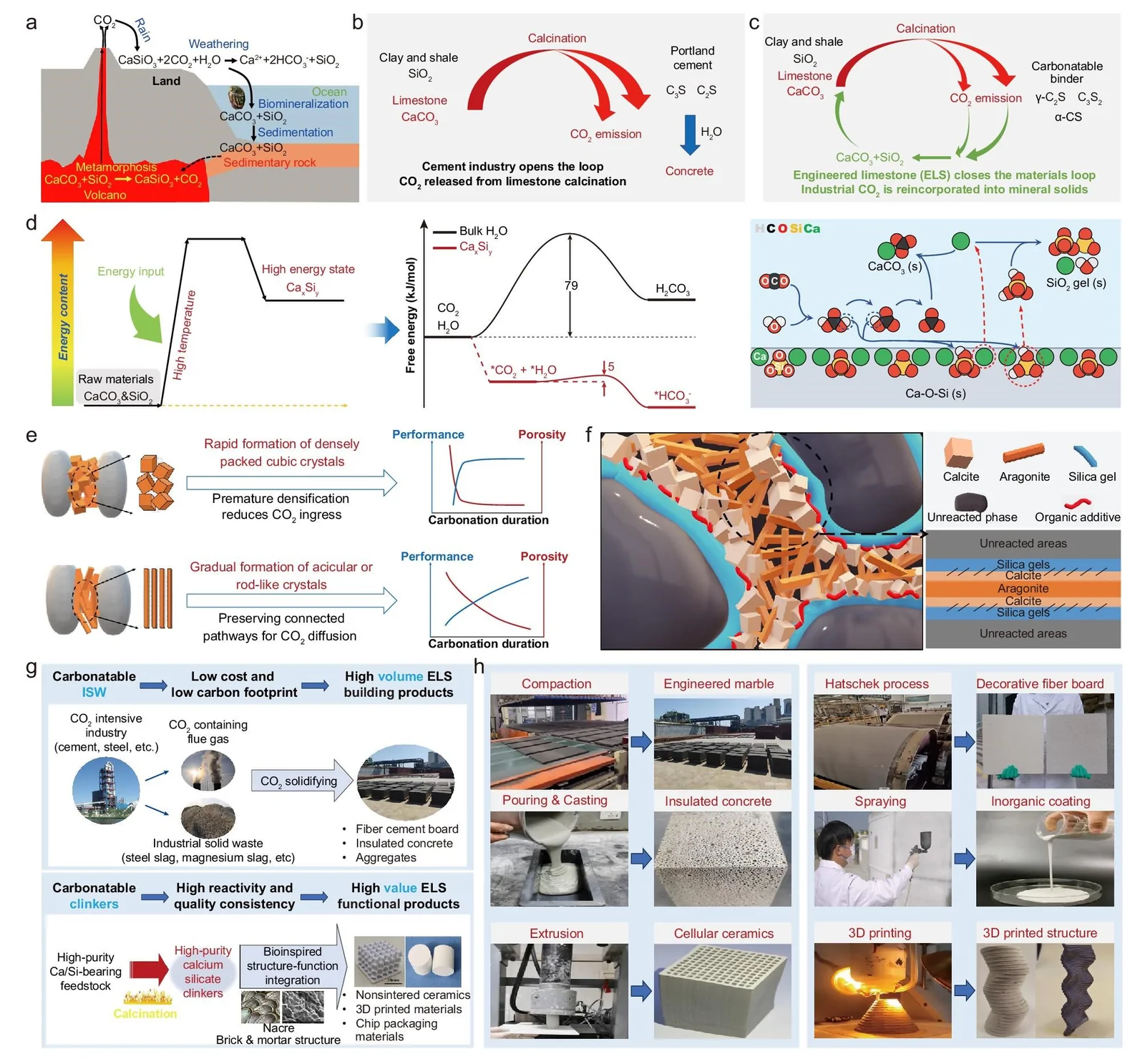
Conceptual framework of ELS for closing the carbon loop through geo- and bio-inspired mineralization. (a–c) Carbon-loop concept: natural calcium-silicate cycling stores CO_2_ as limestone over geological timescales, whereas cement production reverses this process by calcining limestone and releasing CO_2_; ELS closes the materials loop by reincorporating industrial CO_2_ into carbonate-rich construction materials. (d) Kinetic basis of ELS formation, where high-energy silicates and interfacial CO_2_ activation overcome slow silicate dissolution and accelerate coupled dissolution–precipitation. (e and f) Microstructure design principle: calcite-rich regions provide early strength, aragonite-rich networks preserve CO_2_ transport pathways and improve toughness, and silica gel bonds carbonate phases into a cohesive matrix; (f) is reproduced from Ref. [9] with permission from the Chinese Ceramic Society, Copyright 2023. (g) Two application pathways based on low-cost carbonatable wastes for high-volume products and designed clinkers for high-value functional materials. (h) Representative processing technologies for manufacturing ELS products.

In this Perspective, engineered limestone (ELS) is defined as a CO_2_-cured carbonate-silicate composite produced through a deliberately designed engineered carbonate-silicate (ECS) pathway, in which reactive calcium/magnesium silicate clinkers or alkaline industrial solid wastes consume CO_2_ and form a carbonate-rich, stone-like matrix. The ECS pathway begins with the synthesis or selection of highly CO_2_-reactive precursors (e.g. γ-C_2_S, C_3_S_2_, CS, or some alkaline industrial solid wastes rich in these phases), a step that parallels the calcination stage of cement production but is directed toward subsequent CO_2_ uptake. These binders are designed to react rapidly with CO_2_ under mild conditions to generate ELS (Fig. 1c) [3]. Significantly, this ECS pathway transforms CO_2_ from being a flue gas pollutant to a primary reagent for material synthesis, thereby connecting carbon-intensive industries with the production of durable, carbonate-rich construction materials. This definition distinguishes ELS from conventional carbonation-cured concrete or isolated mineralized aggregates: its essential features are a carbonatable silicate feedstock, CO_2_ as a hardening and mineralization reagent, and the formation of a carbonate-silica gel matrix. The framework is based on previous studies of pure calcium silicate clinkers, waste-derived carbonatable binders, artificial stone, boards, coatings, 3D-printed components, and bio-inspired mineralized composites.

The key to preparing ELS is overcoming the rate-limiting step in conventional mineral carbonation, namely the slow dissolution of CO_2_ and calcium silicate minerals and the insufficient supply of carbonate species at reactive surfaces [4]. The newly created ECS pathway addresses this limitation by using thermodynamically high-energy silicates, such as γ-C_2_S and C_3_S_2_, whose metastable crystal structures and elevated synthesis enthalpies (e.g. $\Delta H_r^\circ \ $of γ-C_2_S is 219.9 kJ/mol, ∼2.5 times that of natural wollastonite) make them more reactive with CO_2_ than natural silicates such as wollastonite (Fig. 1d). This thermodynamic instability facilitates the formation of a highly reactive solid–liquid interface, where adsorbed CO_2_ and carbon species promote dissolution. Specifically, the surface-adsorbed CO_2_ is electronically activated, which markedly lowers the activation barrier for its hydration and dissolution [4,5]. Furthermore, the resulting proton release accelerates Ca^2+^ leaching from the silicate lattice, while the locally enriched carbonate species immediately precipitate with dissolved cations to form calcium carbonate polymorphs. The coupled dissolution–precipitation process continuously consumes reaction products, maintains chemical gradients, and reduces surface passivation, thereby sustaining carbonation under ambient pressure and near-room-temperature conditions. Consequently, under controlled curing and transport conditions, even low-concentration industrial flue gas can serve directly as a curing reagent, converting reactive silicate binders into a consolidated carbonate-silica gel matrix over an industrially practical timescale.

Microstructure formation in ELS is governed by distinct roles of calcium carbonate polymorphs in controlling early consolidation, CO_2_ transport and damage resistance [6,7]. Calcite typically forms readily during carbonation as densely packed rhombohedral or near-cubic crystals. This morphology promotes tight particle packing, reduces pore volume and provides early strength. However, a microstructure dominated by compact calcite may block gas-transport pathways, restricting further CO_2_ ingress and preventing the sustained progress of carbonation. Aragonite can play a complementary role: its acicular or rod-like crystals can grow into interlocked networks that reinforce the matrix while preserving connected pathways for CO_2_ diffusion, thereby supporting deeper penetration of CO_2_ and progressive strength gain (Fig. 1e). These functions are not intrinsic or fixed assignments for each polymorph; rather, the contribution of a given CaCO_3_ phase depends on the polymorph assemblage formed under specific solution chemistry, additives, temperature, supersaturation, and curing conditions. The accompanying silica-rich gel is equally important. As calcium is leached from the silicate precursor, the residual silica phase reorganizes into an amorphous gel that fills nanoscale pores and bonds with adjacent carbonate crystals, an event that introduces additional interfaces for crack deflection and energy dissipation [8]. Thus, compressive strength, toughness, CO_2_ transport, and the durability of ELS are governed not simply by the total amount of carbonate formed, but by the spatial arrangement of calcite, aragonite, and silica gel.

Consideration of these coupled effects suggests that an ideal hierarchical ELS architecture (Fig. 1f) would consist of a calcite-rich inner skeleton to provide compressive strength, an aragonite-rich outer or interparticle network that maintains permeability and improves toughness, and a continuous silica-gel phase that integrates the carbonate phases into a cohesive carbonate–silicate matrix [9]. Among possible candidates, γ-C_2_S most closely matches this model for assembling an ideal carbonated matrix in ELS. This substance combines excellent CO_2_ reactivity with negligible hydration reactivity, minimizing interference from hydration products and favoring long-term phase stability. Its unique self-pulverization behavior can also reduce or eliminate energy-demanding grinding during production [10]. The emphasis on γ-C_2_S does not exclude the potential use of other carbonatable phases. C_3_S_2_, CS, or industrial solid wastes rich in these phases (such as steel slag, magnesium slag, and phosphorus slag) might be preferable when processing cost, local availability, phase purity, curing conditions, or product requirements are different.

In optimized systems, ELS can undergo structural consolidation within 24 h under industrially relevant curing conditions through a mineralization process that resembles the formation of natural carbonate-based materials such as limestone. Compression of the geological timescale (millennia) into an industrially viable timeline is achieved by engineering the reactivity of precursors and microstructural properties. The nature-inspired features of ELS suggest two distinct application pathways (Fig. 1g). The first is a high-volume route for building products, for which the main evaluation criteria are scale, cost, feedstock availability, and CO_2_ utilization. Here, alkaline industrial wastes, such as steel slag, magnesium slag, and phosphorus slag, serve as carbonatable feedstocks [11]. Representative products include decorative artificial stone, which mimics aspects of natural marble formation while greatly shortening the preparation timescale and operating under much milder conditions, and fiber cement boards, where conventional autoclaved curing can be replaced by ambient-temperature and ambient-pressure CO_2_ curing that simultaneously hardens the material and stores CO_2_ [12,13]. The second pathway is driven by product value, where the main criteria are microstructural precision, function, and performance. The approach uses more controlled clinkers obtained in a process similar to cement manufacturing, where phase purity, reactivity, and microstructure justify higher processing costs. Drawing inspiration from natural biominerals (e.g. nacre, coral) and metamorphic rocks (e.g. marble), ELS leverages advanced microstructural engineering in the carbonated matrix of this high-purity clinker. This biomimetic approach allows the replication of evolutionary-optimized structures through ambient-temperature processing, a distinct advantage over those required for manufacturing. This route supports performance-led products, such as 3D-printed materials with strengthened interlayers, dense carbon-mineralized ceramics, durable inorganic coatings, and bioinspired nacre-like composites [14–16]. The examples, demonstrated through laboratory compacts, boards, artificial stone, coatings, and 3D-printed prototypes, illustrate that ELS is a platform rather than a single product. Six tailored processing technologies further show how CO_2_ can be transformed into construction and functional materials (Fig. 1h).

Several scientific hurdles remain in validating the potential of ELS. Durability must be assessed for wetting-drying, freeze-thaw, chloride exposure, and abrasion using protocols appropriate to carbonate-rich matrices rather than relying solely on Portland cement concrete tests. Manufacturing also requires better control of feedstock variability, heavy-metal immobilization, gas transport through larger components, and curing uniformity. These issues determine whether the attractive reaction kinetics observed in powders, compacts and prototypes can be translated into robust products.

Carbon accounting and standardization form a second set of challenges. Claims of carbon negativity should remain case-specific until lifecycle assessment includes precursor calcination, grinding or self-pulverization, transport, CO_2_ source and conditioning, curing energy, and service life. ELS products also need performance-based specifications for carbonate–silicate binders, waste-derived feedstocks, and CO_2_ curing that do not fit neatly into existing Portland cement categories. ELS will not replace cement universally, nor should it be represented as a general solution to carbon capture. Its practical role is narrower but important: converting selected calcium-rich resources into useful construction materials by making industrial CO_2_ a hardening and mineralization reagent. This is the practical meaning of closing the carbon loop for sustainable construction.
